# Moderating effects of body composition biomarkers on the relationship between thyroid hormones and cognitive performance in euthyroid older adults: insights from NHANES data

**DOI:** 10.3389/fendo.2024.1487614

**Published:** 2024-11-21

**Authors:** Xiaosong Li, Hongliang Duan, Shuang Liu, Hanyang Li, Hong Zhang

**Affiliations:** Department of Thyroid Surgery, The Second Hospital of Jilin University, Changchun, Jilin, China

**Keywords:** thyroid hormones, cognitive function, memory, body composition, waist circumstance

## Abstract

**Background:**

Thyroid hormones are essential for cognitive function and can impact cognitive performance even in euthyroid individuals. This study investigates how thyroid hormones influence cognitive performance in the elderly and whether body composition biomarkers moderate this relationship. The aim is to determine if lifestyle interventions should prioritize weight loss, overall body fat reduction, or abdominal fat loss.

**Methods:**

We analyzed data from the NHANES 2011-2012 dataset, focusing on thyroid hormone levels, cognitive performance, and body composition metrics in euthyroid individuals aged 60 to 80 years. A total of 573 participants were included in the analysis. Pearson correlation analyses were conducted to evaluate the associations between thyroid hormone indicators and cognitive performance metrics. Ordinal logistic regression and linear regression analyses were used to determine the predictive capacity of thyroid hormones on cognitive functions, adjusting for potential confounders such as age, gender, and education level. Statistical analyses were performed using R Studio and Stata, utilizing Pearson correlation, ordinal logistic regression, and linear regression methods.

**Results:**

Significant correlations were observed between short-term memory and TT3 (*r =* 0.111, *p =* 0.018), TSHI (*r =* -0.121, *p =* 0.010), and TFQI (*r =* -0.107, *p =* 0.023); delayed memory and FT3 (*r =* 0.143, *p =* 0.003), TT3 (*r =* 0.146, *p =* 0.002), and TSHI (*r =* -0.125, *p =* 0.009); and executive function with FT4 (*r =* -0.141, *p =* 0.003) and the FT3/FT4 ratio (*r =* 0.137, *p =* 0.004). Although thyroid indicators did not independently predict short-term memory (OR = 0.006, *p =* 0.116), they were statistically significant for delayed memory with FT3 (OR = 0.642, *p =* 0.017) and TT3 (OR = 0.010, *p =* 0.015). Linear regression analysis indicated that FT4 (t = -2.99, *p =* 0.003) and the FT3/FT4 ratio (t = 2.91, *p =* 0.004) were significant predictors of executive function. Hierarchical regression analyses revealed that BMI and waist circumference (WWI) significantly moderated the relationship between thyroid function and short-term memory (BMI: z = 2.44, *p =* 0.015; WWI: z = -2.19, *p =* 0.029). BMI also moderated the models for delayed memory (z = 2.11, *p =* 0.035), while RFM and C-index did not exhibit significant moderating effects. No moderators were identified in the relationship between executive function and thyroid hormones.

**Conclusion:**

This study underscores the significant influence of higher BMI and waist circumference on the relationship between thyroid function and memory performance. In contrast, body composition indicators such as RFM and C-index do not appear to significantly affect cognitive function related to thyroid levels, highlighting the importance of fat distribution in cognitive health assessments.

## 1 Introduction

Thyroid hormones are essential for maintaining cognitive function, with both hypothyroidism and hyperthyroidism being associated with cognitive impairment (1–5). Even among individuals with normal thyroid function, fluctuations in hormone levels can influence cognitive performance. Specifically, lower levels of free triiodothyronine (FT3) and higher levels of free thyroxine (FT4) within the normal range have been linked to an increased risk of Alzheimer’s disease and dementia, respectively (6, 7). Additionally, lower thyroid-stimulating hormone (TSH) levels within the normal range have been identified as an independent risk factor for mild cognitive impairment and Alzheimer’s disease (8, 9). Brain imaging studies further highlight significant differences in critical cognitive regions between individuals with thyroid dysfunction and those with euthyroidism (10). However, a meta-analysis of 23 cohort studies found no strong association between thyroid function and cognitive performance or dementia risk, although a trend of lower cognitive function was observed in patients with overt hypothyroidism (11). These inconsistent findings may be due to the exclusive use of serum FT3, FT4, and TSH measurements, which may not fully capture thyroid hormone sensitivity. Therefore, utilizing data from the National Health and Nutrition Examination Survey (NHANES) (12), this study first aims to investigate the relationship between thyroid hormones, including sensitivity indicators, and cognitive outcomes.

Considering that obesity is a major risk factor for thyroid-related diseases (13), our study incorporates various body composition biomarkers as covariates to explore their potential influence on this relationship. Given that current treatments for thyroid diseases, such as hormone replacement therapy, often fail to reverse cognitive impairment due to the low bioavailability of exogenous thyroid hormones in the central nervous system (3), non-pharmacological approaches may offer a viable alternative. By examining the moderating effects of body composition on the relationship between thyroid hormones and cognitive performance, our second aim is to provide insights into whether lifestyle interventions should prioritize overall weight loss, fat reduction, or specifically abdominal fat reduction.

## 2 Methods

### 2.1 Data source

This study utilized data from the NHANES 2011-2012 (12). NHANES is an ongoing survey program conducted by the National Center for Health Statistics (NCHS), a branch of the Centers for Disease Control and Prevention, aimed at assessing the health and nutritional status of the U.S. population. NHANES employs a multistage sampling design to draw a representative sample from the civilian noninstitutional population. Data collection methods include interviews, physical examinations, and laboratory tests, covering a wide range of health information. The data collected includes, but is not limited to, demographic information (e.g., age, sex, race/ethnicity, education level, income), health behaviors (e.g., dietary habits, physical activity, smoking, and alcohol consumption), health conditions (e.g., chronic disease diagnoses and self-reported health status), biological measurements (e.g., blood pressure, weight, lipids, glucose), and laboratory tests (e.g., blood and urine sample analyses). The 2011-2012 NHANES dataset includes information from 9,756 respondents, with this study focusing on demographic data, thyroid function indicators, body composition and circumferential measurements, and cognitive function test results. The NHANES data collection and use adhere strictly to ethical standards and privacy protection measures. Participants provided informed consent before participating in the survey, and all data were anonymized to protect the privacy of the respondents. The data used in this study are publicly available, downloaded from the NCHS website, and comply with relevant data usage policies and ethical requirements.

### 2.2 Measurements

#### 2.2.1 Thyroid hormone

Thyroid hormone levels were assessed using eight thyroid function indicators provided in the 2011-2012 NHANES data: FT3, FT4, total triiodothyronine (TT3), total thyroxine (TT4), thyroglobulin (TG), thyroglobulin antibodies (TGAb), TSH, and thyroid peroxidase antibodies (TPOAb). Additionally, four thyroid hormone sensitivity indicators were calculated using specific formulas: FT3/FT4 ratio, Thyroid Feedback Quantile-based Index (TFQI), Jostel’s TSH index (TSHI), and thyrotropin thyroxine resistance index (TT4RI). The definitions and formulas for these sensitivity indicators are detailed in the **Supplementary Materials**. Initially, all the indicators were identified as numerical variables; However, due to violations of assumptions for numerical data, some were subsequently categorized and grouped by quartiles.

#### 2.2.2 Cognitive function

Cognitive function was assessed using three tests from the NHANES 2011-2012 data, focusing on memory (including short-term and delayed memory), executive function, and working memory.

- Memory: Memory was evaluated using the Consortium to Establish a Registry for Alzheimer’s Disease Word Learning subtest (14). This test assesses short-term and delayed memory of new words. Participants were asked to read and memory a list of ten unrelated words across three consecutive trials, followed by a delayed memory test after completing the other cognitive assessments. The number of correctly memoryed words in each trial was recorded. Depending on the distribution of results, these data were treated as either categorical or numerical variables.
- Executive Function: Executive function was measured using the Animal Fluency test (15). In this test, participants were asked to name as many animals as possible within one minute. The total number of correctly named animals was recorded as a numerical variable.
- Working Memory: Working memory was assessed using the Digit Symbol Substitution Test (16), which is derived from the Wechsler Adult Intelligence Scale (17). Participants were required to match symbols with their corresponding numbers within a two-minute time frame, with the score being the total number of correct matches. This score was considered a numerical variable.

#### 2.2.3 Body composition

Body composition was assessed using height, weight, and waist circumference measurements from the 2011-2012 NHANES data, along with sex information, to calculate four body composition indicators: Body mass index (BMI), Conicity index (C-index), Relative fat mass (RFM), and Weight-adjusted waist index (WWI). These indicators provide a comprehensive evaluation of body composition, allowing for the examination of how different types of obesity may moderate the relationship between thyroid function and cognitive function.

- BMI was calculated using weight and height to reflect overall obesity $\left(BMI=\;\frac{\text{weight}\;\left(kg\right)\;}{\text{height}\;\;(m^{2})\;}\right)$ , and it is widely used in clinical practice and research (18).
- C-index measures central obesity by assessing the ratio of waist circumference to height, better reflecting visceral fat distribution $C\;index=\;\frac{\text{waist}\;\left(m\right)\;}{0.109\sqrt{\frac{\text{weight }\left(\text{kg}\right)\;}{\text{height }\left(m\right)\;}}}$ (19).
- WWI adjusts waist circumference for weight to assess central fat distribution more accurately ( $WWI=\;\frac{\text{waist}\;\left(cm\right)\;}{\sqrt{\text{weight}\;\left(kg\right)\;}}$ ) (18).
- RFM estimates relative fat mass using height, waist circumference, and sex (19). The formulas are as follows: $\text{Male }RFM\;=64-\left[20\times \left(\frac{\text{height}\;\left(m\right)}{\text{waist}\;\left(m\right)}\right)\right],\text{ Female }RFM\;=70-\left[20\times \left(\frac{\text{height}\;\left(m\right)\;}{\text{waist }\left(m\right)\;}\right)\right]\;$ (19).

### 2.3 Statistical analysis

The NHANES data underwent an initial cleaning and verification process, including tests for normality and inspections of bivariate scatterplots. Outliers and extreme values were managed using mean substitution, while skewed data were categorized into quartiles or quintiles and treated as categorical variables. Statistical analyses were conducted in accordance with standard assumptions. For variables meeting the necessary numerical criteria, Pearson correlation analyses were employed to assess linear relationships and to preliminarily screen variables for inclusion in subsequent regression analyses. Ordinal logistic regression and linear regression models, adjusted for age, sex, and education, were then utilized to investigate the effects of thyroid function on cognitive function, as well as the moderating role of body composition indicators. All statistical analyses and visualizations were carried out using R Studio 4.2.2 (20) and Stata 17 (21). The significance level was set at.05.

## 3 Results

### 3.1 Participant demographics

The demographic characteristics, including age, race, family poverty index, education level, and BMI range, were summarized by gender for the participants included in the study (*n* = 573). Details are provided in **Table 1**.

**Table 1:** Demographic Information.

	Male (*n* = 285)	Female (*n* = 288)
Age (*n* = 573)		
60-65	103	107
66-70	57	53
71-75	49	42
76-80	76	86
Race (*n* = 573)		
Mexican American	18	22
Other Hispanic	31	33
Non-Hispanic White	111	121
Non-Hispanic Black	84	80
Other race (including multi-racial)	41	32
Ratio of family income to poverty (*n* = 571)		
<0.5	11	14
0.5 - < 1.0	37	48
1.0 - < 2.0	82	67
>2.0	153	159
Education level (*n* = 571)		
Less than 9^th^ grade	54	51
9-11th grade (Includes 12th grade with no diploma)	50	58
High school graduate/GED or equivalent	52	55
Some college or AA degree	63	84
College graduate or above	65	39
BMI range (*n* = 554)		
Underweight (<18.5)	3	8
Healthy weight (18.5 ~ 24.9)	89	79
Overweight (25 ~ 29.9)	110	82
Class I obesity (30 ~ 34.9)	49	65
Class II obesity (35 ~ 39.9)	12	26
Class III obesity (>40)	14	17

A family poverty index (ratio of family income to poverty) >1 is defined as non-poor, while <1 is defined as poor. BMI, body mass index.

### 3.2 Correlations between thyroid hormones and cognitive performance

**Figure 1:**
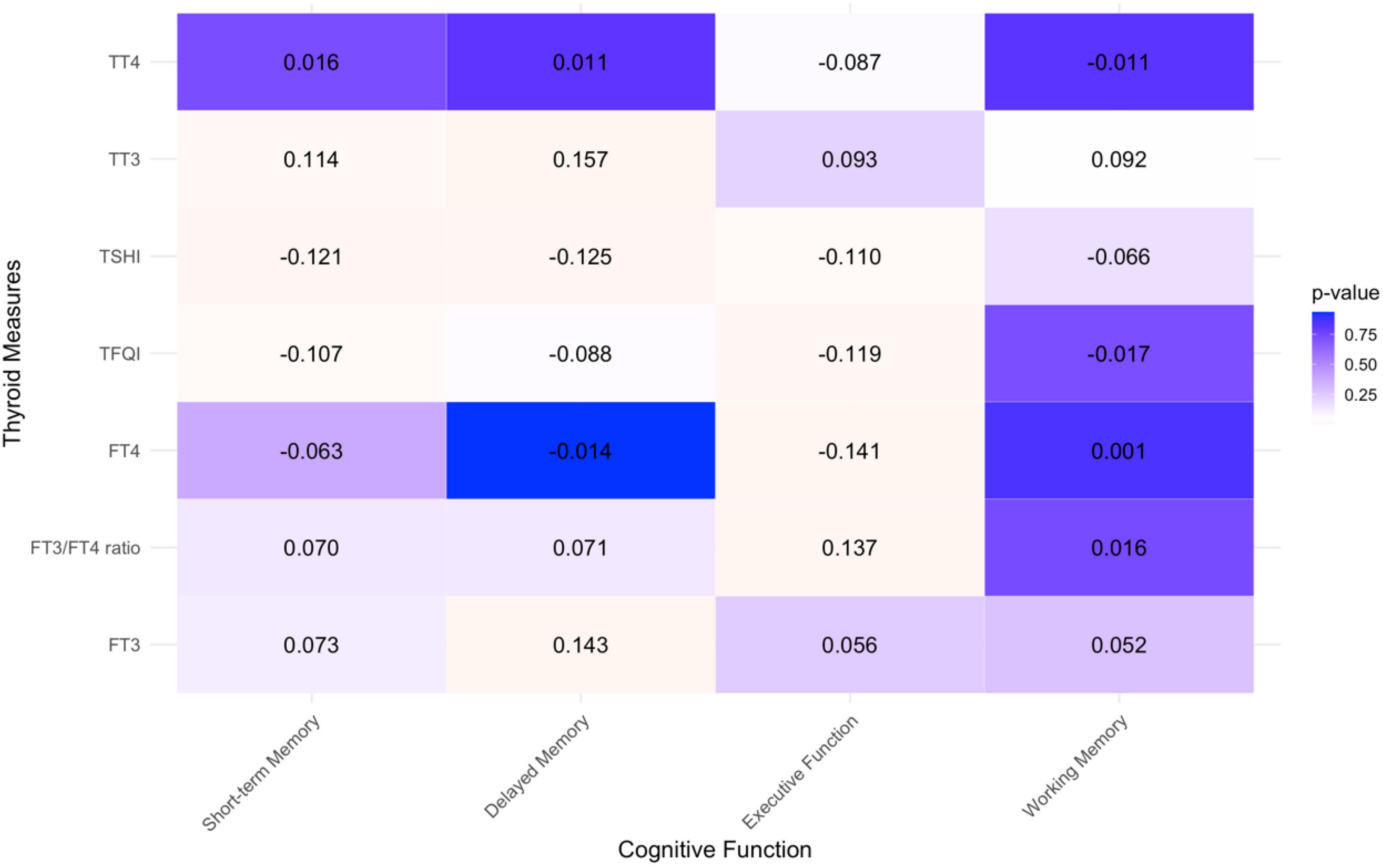
Heat map depicting correlations between thyroid hormones and cognitive function.

A Pearson correlation analysis was conducted to explore the linear relationship between thyroid function indicators (FT3, FT4, TT3, TT4, FT3/FT4 ratio, TSHI, and TFQI) and cognitive performance indicators that met the data distribution requirements (see **Figure 1**). Among these, short-term memory showed a significant linear correlation with TT3 (*r =* 0. 111, *p =* 0.018), TSHI (*r =* -0.121, *p =* 0.010), and TFQI (*r =* -0.107, *p =* 0.023); delayed memory had significant linear correlations with FT3 (*r =* 0.143, *p =* 0.003), TT3 (*r =* 0. 146, *p =* 0.002), and TSHI (*r =* -0.125, *p =* 0.009); and executive function was significantly correlated with FT4 (*r =* -0.141, *p =* 0.003), the FT3/FT4 ratio (*r =* 0.137, *p =* 0.004), TSHI (*r =* -0.108, *p =* 0.023), and TFQI (*r =* -0.119, *p =* 0.012). There were no significant linear correlations between working memory and any of the thyroid function indicators. Since all data were approximately normally distributed and no other trends were observed upon examining scatter plots, it is assumed that the association between working memory and thyroid function is weak, and thus, working memory was not included in the subsequent analyses.

### 3.3 Regression analysis of thyroid hormones predicting cognitive function

Ordinal logistic regression and linear regression analyses were conducted for short-term memory with TT3, TSHI, and TFQI, delayed memory with FT3, TT3, and TSHI, and executive function with FT4, FT3/FT4 ratio, TSHI, and TFQI, all of which showed significant correlations.

Using ordinal logistic regression, we examined the predictive effects of TT3 (**Supplementary Table S1**), TSHI (**Supplementary Table S2**), and TFQI (**Supplementary Table S3**) on short-term memory. After adjusting for confounding variables, including age, gender, and education level, all three regression models remained statistically significant: χ^2^ (7, *N* = 452) = 84.55, *p =* 0.001, pseudo *R* ^2^ = 0.047; χ^2^ (7, *N* = 452) = 83.38, *p =* 0.001, pseudo *R* ^2^ = 0.046; χ^2^ (7, *N* = 452) = 83.28, *p =* 0.001, pseudo *R* ^2^ = 0.046. However, the effects of the three thyroid hormone indicators on the outcome variable, short-term memory, were not statistically significant: *OR* = 0.006, 95% confidence interval (*CI*) [-0.002, 0.014], *p =* 0.116; *OR* = -0.141, 95% *CI* [-0.382, 1.000], *p =* 0.251; *OR* = -0.249, 95% *CI* [-0.690, 0.193], *p =* 0.269. These findings suggest that thyroid hormone levels do not independently predict short-term memory.

We also examined the predictive effects of FT3 (**Supplementary Table S4**), TT3 (**Supplementary Table S5**), and TSHI (**Supplementary Table S6**) on delayed memory by ordinal logistic regression. After controlling for confounding variables, the results from multivariate ordinal logistic regressions indicated that all three regression models remained statistically significant: χ^2^ (7, *N* = 444) = 115.61, *p =* 0.001, pseudo *R* ^2^ = 0.059; χ^2^ (7, *N* = 444) = 115.88, *p =* 0.001, pseudo *R* ^2^ = 0.059; χ^2^ (7, *N* = 444) = 111.14, *p =* 0.001, pseudo *R* ^2^ = 0.057. Among these, the effects of FT3 and TT3 on the outcome variable, delayed memory, were statistically significant: *OR* = 0.642, 95% *CI* [0.117, 1.166], *p =* 0.017; *OR* = 0.010, 95% *CI* [0.002, 0.019], *p =* 0.015. However, the effect of TSHI on delayed memory was not statistically significant: *OR* = -0.138, 95% *CI* [-0.373, 0.096], *p =* 0.248.

We conducted linear regression analyses to examine the relationship between FT4 (**Supplementary Table S7**), FT3/FT4 ratio (**Supplementary Table S8**), TSHI (**Supplementary Table S9**), and TFQI (**Supplementary Table S10**; which meet the basic assumptions of linear regression) and executive function. The univariate linear regression results showed that all four thyroid function indicators [i.e., FT4 (*t =* -2.99, *p =* 0.003), FT3/FT4 ratio (*t =* 2.91, *p =* 0.004), TSHI (*t =* -2.29, *p =* 0.023), and TFQI (*t =* -2.52, *p =* 0.012)] had significant predictive effects on executive function, with significant regression models: *F*(1,443) = 8.91, *p =* 0.003, adjusted *R* ^2^ = 0.018; *F*(1,443) = 8.46, *p =* 0.004, adjusted *R* ^2^ = 0.017; *F*(1,443) = 5.24, *p =* 0.023, adjusted *R* ^2^ = 0.010; and *F*(1,443) = 6.37, *p =* 0.012, adjusted *R* ^2^ = 0.020. After controlling for confounding factors, FT4, FT3/FT4 ratio, and TFQI remained statistically significant predictors in the multiple linear regression results: *t =* -2.70, *p =* 0.007; *t =* 2.24, *p =* 0.025; *t =* -1.99, *p =* 0.047. The overall regression models were significant: *F*(7,442) = 16.20, *p =* 0.001, adjusted *R* ^2^ = 0.194; *F*(7,434) = 15.81, *p =* 0.001, adjusted *R* ^2^ = 0.190; *F*(7,442) = 15.62, *p =* 0.001, adjusted *R* ^2^ = 0.188. Although the regression model for TSHI and executive function was significant, *F*(7,442) = 15.31, *p =* 0.001, adjusted *R* ^2^ = 0.185, TSHI as a predictor was not statistically significant: *t =* -1.490, *p =* 0.137.

### 3.4 Moderating effects of body composition on the thyroid-cognition relationship

We used hierarchical regression analysis to examine the moderating effects of various biological body composition indicators—BMI, RFM (categorized into quintiles), C-index, and WWI—on the relationship between thyroid function and cognitive performance.

**Figure 2:**
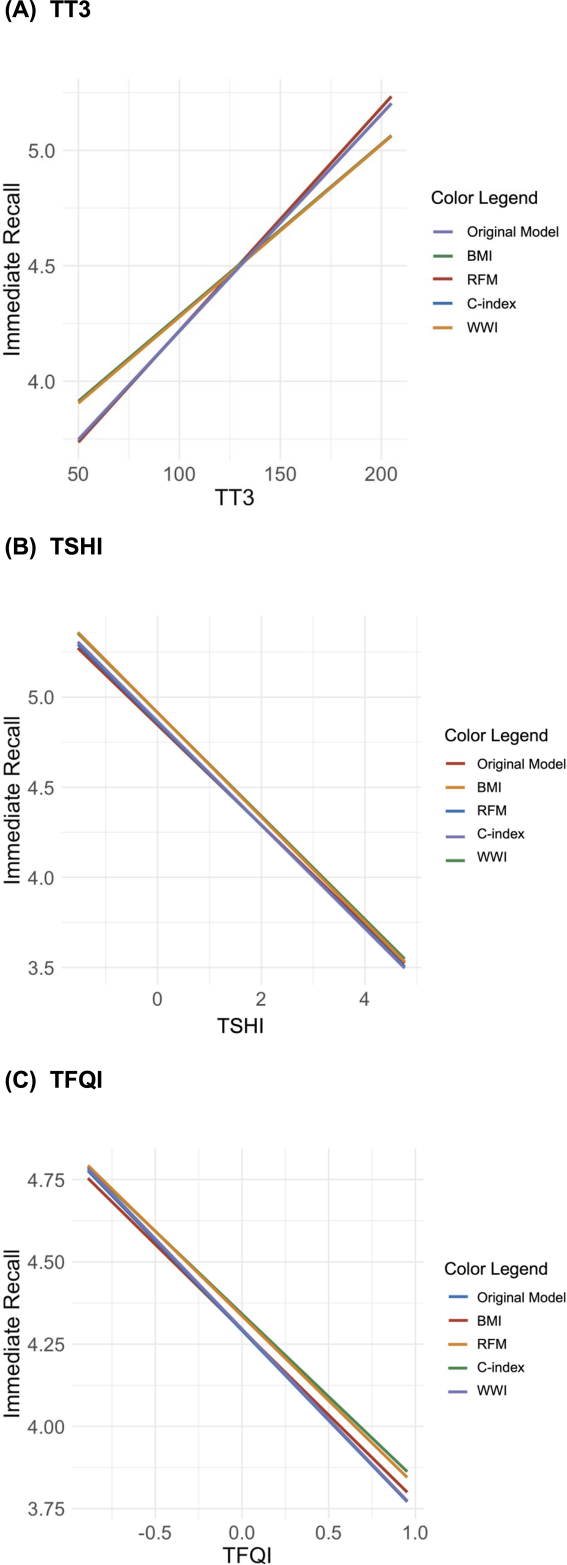
Interaction plots of the moderating effects of body composition on the relationship between short-term memory and thyroid hormones [**(A)** total triiodothyronine (TT3), **(B)** Jostel’s TSH index (TSHI), and **(C)** thyroid feedback quantile-based index (TFQI)].

The analysis revealed that BMI and WWI significantly moderated the models predicting short-term memory based on thyroid function indicators (TT3, TSHI, and TFQI; **Figure 2**). For BMI, the standardized slopes in these models were significant, with z-values of 2.44 (*p =* 0.015), 2.45 (*p =* 0.014), and 2.47 (*p =* 0.014), respectively. The corresponding *R* ^2^ values were 0.050, 0.049, and 0.049 (all *p =* 0.001), indicating robust moderating effects. Similarly, for WWI, the standardized slopes were also significant, with z-values of -2.19 (*p =* 0.029), -2.14 (*p =* 0.032), and -2.09 (*p =* 0.037), and *R* ^2^ values of 0.047, 0.047, and 0.046 (all *p*s = 0.001). Conversely, the moderating effects of the C-index and RFM on these models were not statistically significant. The standardized slopes for the C-index in models involving TT3, TSHI, and TFQI were *z =* 1.32 (*p =* 0.186), *z =* -1.43 (*p =* 0.154), and *z =* -1.29 (*p =* 0.198), respectively, with all models showing an *R* ^2^ of 0.045 (*p =* 0.001). When analyzing RFM across quintiles (using Q1 as a reference), the standardized slopes for Q2 to Q5 did not reach statistical significance for TT3, TSHI, and TFQI. These results suggest that BMI and WWI have significant moderating effects on the relationship between thyroid indicators and short-term memory, whereas RFM and the C-index do not appear to significantly influence these models. Therefore, body weight and waist circumference are more critical factors than relative fat mass and the conicity index in predicting short-term memory based on thyroid function.

**Figure 3:**
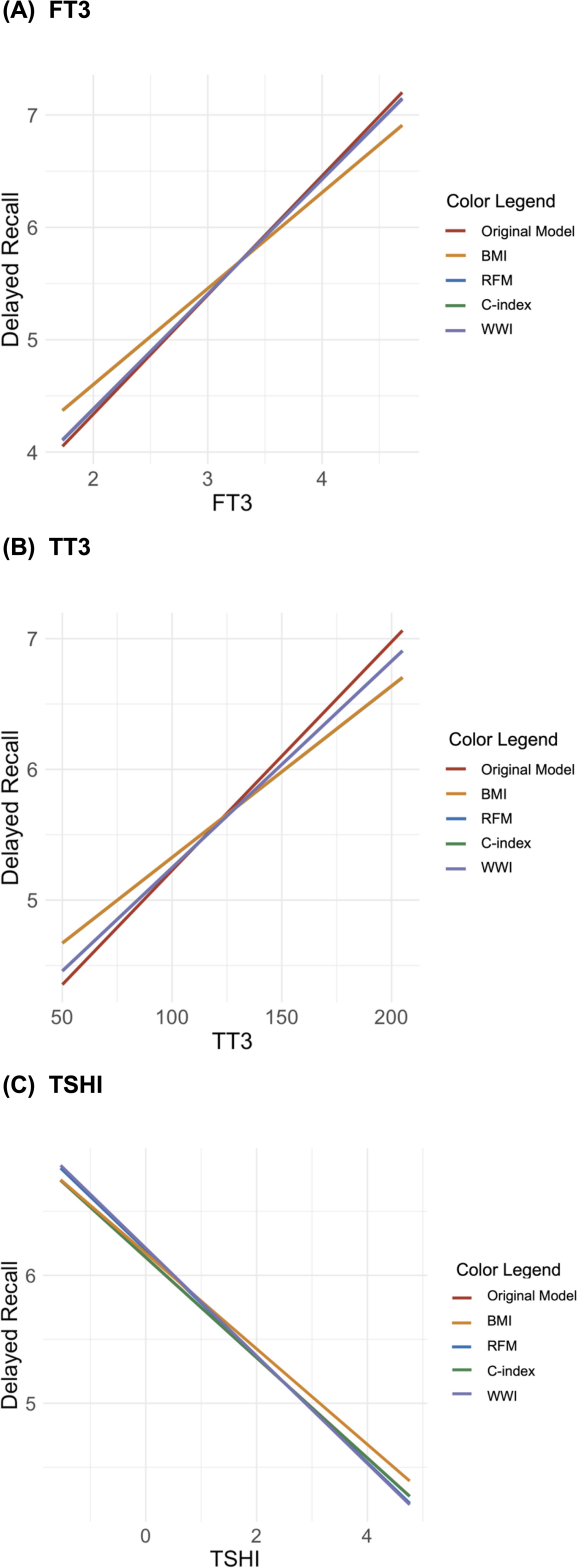
Interaction plots of the moderating effects of body composition on the relationship between delayed memory and thyroid hormones [**(A)** free triiodothyronine, **(B)** total triiodothyronine (TT3), and **(C)** Jostel’s TSH index (TSHI)].

The moderation analysis also indicated that BMI significantly moderates regression models predicting delayed memory for FT3, TT3, and TSHI, but RFM, C-index, and WWI do not (**Figure 3**). The standardized slopes for BMI in these models were *z =* 2.11 (*p =* 0.035), *z =* 2.27 (*p =* 0.023), and *z =* 2.23 (*p =* 0.026), with corresponding *R* ^2^ values of 0.061, 0.060, and 0.059 (*p*s = 0.001). In contrast, the slopes for the C-index, WWI, and RFM (across quintiles) did not achieve statistical significance, demonstrating a limited moderating effect on delayed memory.

**Figure 4:**
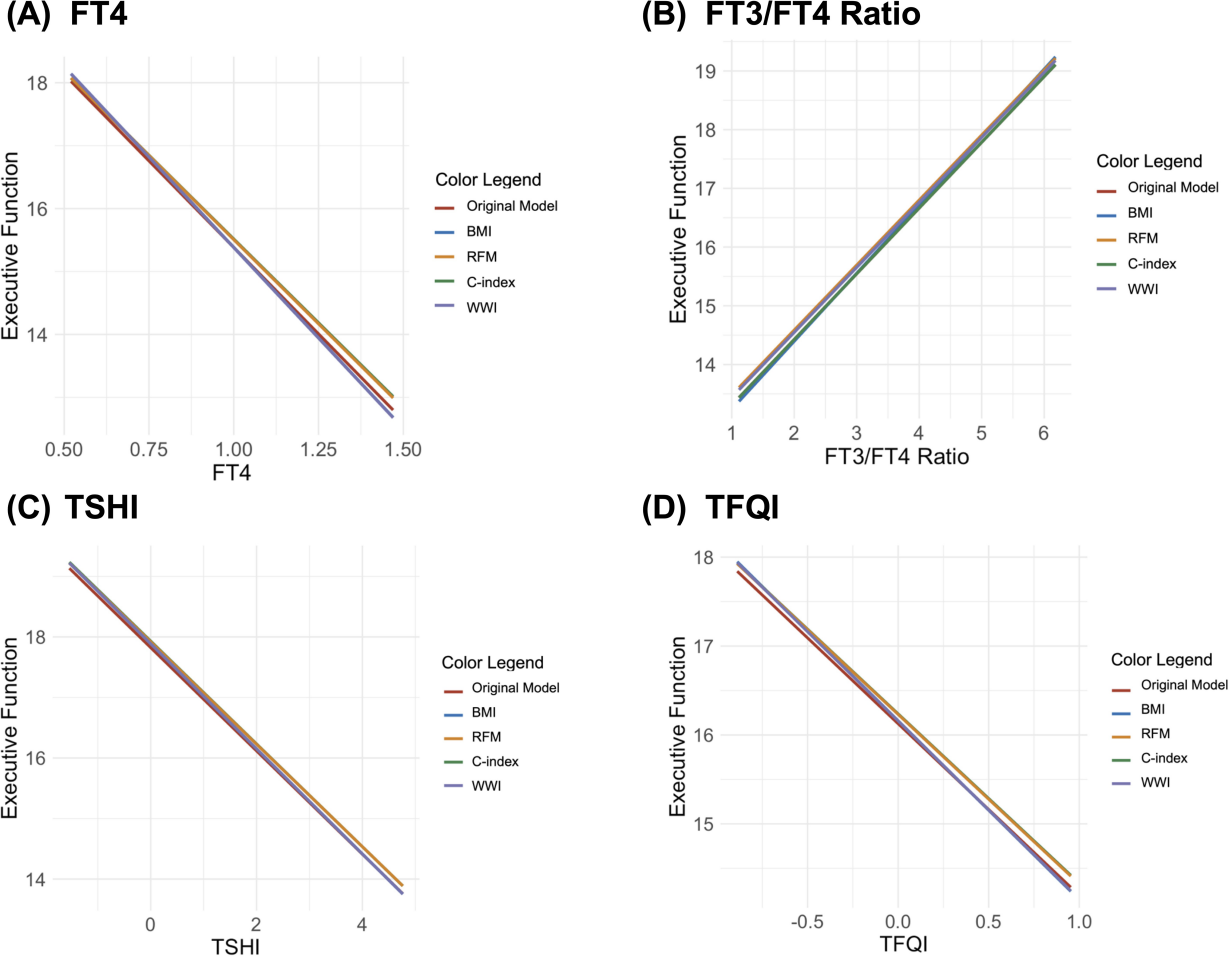
Interaction plots of the moderating effects of body composition on the relationship between executive function and thyroid hormones [**(A)** free thyroxine (FT4), **(B)** tree T3 to tree T4 ratio (FT3/FT4 ratio), **(C)** Jostel’s TSH index (TSHI), and **(D)** thyroid feedback quantile-based index (TFQI)].

Finally, the moderation analysis showed no significant moderating effects of any body composition indicators on the relationship between executive function and thyroid hormones (FT4, FT3/FT4 ratio, TSHI, and TFQI; **Figure 4**). None of the standardized slopes for BMI, RFM, C-index, or WWI were statistically significant in these models. The lack of significant moderating effects indicates that body composition plays a relatively minor role in the relationship between executive function and thyroid function.

Overall, the findings suggest that BMI and WWI are significant moderators in the relationship between thyroid function and memory, while other body composition indicators like RFM and the C-index do not show significant effects. The importance of body weight and waist circumference in cognitive function prediction highlights the need for a more nuanced understanding of the interactions between physical health and cognitive performance.

## 4 Discussion

This study explores the relationship between thyroid hormone levels, including TSH, and cognitive performance in individuals aged 60 to 80 with normal thyroid function, using data from the NHANES 2011-2012 database. We found that thyroid hormone levels are significantly associated with various aspects of cognitive performance, specifically short-term memory, delayed memory, and executive function, though their influence on working memory is less clear. Our results show that certain thyroid hormones, such as TT3, TSHI, and TFQI, correlate with cognitive abilities, with higher levels linked to better performance in short-term memory tests. Both FT3 and TT3 were significant predictors of delayed memory, suggesting that maintaining adequate levels of these hormones may be crucial for effective memory storage and retrieval. While TSH levels were also examined, they showed a less consistent association with cognition. Additionally, body composition indicators, such as BMI and waist circumference, were found to moderate the relationship between thyroid hormones and cognitive performance. Individuals with higher BMI demonstrated different cognitive responses to thyroid hormone levels compared to those with lower BMI, suggesting that body weight and waist circumference may interact with thyroid function, potentially due to metabolic changes that influence cognitive performance.

Interestingly, our study found no significant correlation between TSH levels and cognitive function in our euthyroid cohort, a result that aligns with ongoing debates regarding the reliability of TSH as an indicator of thyroid health. For instance, studies by Ojala et al. (22) and Gan and Pearce (23) have reported mixed findings, indicating that TSH may not serve as a definitive marker of cognitive performance. Ojala et al. (22) observed that higher TSH concentrations were linked to better cognitive scores, although this association was not statistically significant. Meanwhile, Gan and Pearce (23) highlighted the inconsistent evidence surrounding low TSH levels and cognitive impairment. These discrepancies underscore the complexity of the thyroid-cognition relationship and suggest that TSH may not adequately reflect the nuances of thyroid function that influence cognitive outcomes.

The findings suggest that working memory is relatively unaffected by thyroid function. Working memory, which involves the temporary storage and manipulation of information, is a crucial aspect of cognitive function (24). Although thyroid hormones are vital for brain development and function, this study did not find a significant effect of thyroid function on working memory. This observation aligns with previous research suggesting that thyroid hormones may have a limited impact on certain cognitive processes (25). Potential explanations for this include the insensitivity of the neural circuits involved in working memory to variations in thyroid hormone levels, or the existence of compensatory mechanisms that sustain working memory performance despite fluctuations in thyroid hormones (25).

Moreover, this study revealed notable effects of thyroid hormones on both short-term and delayed memory, as well as executive function. TT3, TSHI, and TFQI emerged as statistically robust predictors of short-term memory, highlighting their potential as indicators for this cognitive domain. This finding corroborates previous research suggesting that fluctuations in TT3 levels can influence short-term information processing and storage (26). For delayed memory, FT3, TT3, and TSHI demonstrated significant predictive effects, indicating that delayed memory may rely more on a continuous supply of thyroid hormones (6, 27). Variations in FT3 and TT3 levels are believed to impact brain regions involved in memory storage and retrieval, thereby influencing memory performance. Additionally, FT4, the FT3/FT4 ratio, TSHI, and TFQI were significant predictors of executive function, which encompasses higher-order cognitive processes such as planning, decision-making, and problem-solving (2, 28). This suggests that thyroid hormones may play a crucial role in regulating these higher-order functions.

The study also examined the moderating role of body composition biomarkers in the relationship between thyroid hormones and cognitive function. Body weight was found to significantly moderate both short-term and delayed memory, possibly due to the influence of metabolic and hormonal changes on thyroid function and the demands of long-term information storage and retrieval (2). Waist circumference moderated the effect of thyroid function on short-term memory, highlighting its role in metabolic health and, consequently, cognitive performance (19). Although executive function was most strongly associated with thyroid function, the moderating effect of body composition indicators, including weight and waist circumference, was minimal. The core aspects of executive function, such as planning, organization, flexibility, and inhibitory control, may be particularly sensitive to thyroid hormones (29). Despite the importance of body composition for overall health, it did not significantly influence the relationship between thyroid function and executive function in this study, suggesting that thyroid hormones may independently exert a more substantial impact on executive function.

This study presents several notable strengths. First, the data were obtained from a national health and nutrition survey in the United States, which ensures high data quality, a large sample size, and extensive demographic coverage (12). Second, while previous research has primarily examined the relationship between traditional thyroid hormone markers such as FT3, FT4, and TSH and cognitive function, this study uniquely incorporates thyroid hormone sensitivity markers (7, 28). Additionally, in light of the ongoing debate regarding the efficacy of various body composition indicators (19), this study goes beyond BMI by including three emerging indicators to comprehensively assess the moderating role of body composition in the relationship between thyroid hormones and cognitive performance.

Despite these strengths, the study has several limitations. Although age, sex, and education were controlled for as confounding factors, the presence of other unaccounted or unknown confounders may have introduced information bias. Second, the study population was restricted to U.S. residents participating in the NHANES cohort. While this cohort included individuals from diverse ethnic backgrounds, the findings may have limited generalizability on a global scale, necessitating further validation of the results. Additionally, due to the observational nature of the study, it can only establish associations rather than causal relationships (30). Finally, the original dataset contained a substantial amount of missing and extreme values, which resulted in a low sample retention rate following the application of inclusion and exclusion criteria.

## 5 Conclusion

In conclusion, this study provides significant evidence of the relationship between thyroid hormones and cognitive function in older adults, as well as the moderating effects of body composition. While working memory appears minimally affected by thyroid function, short-term memory, delayed memory, and executive function show strong associations with various thyroid hormone indicators. Specifically, TT3, TSHI, and TFQI are linked to short-term memory; FT3, TT3, and TSHI are associated with delayed memory; and FT4, the FT3/FT4 ratio, TSHI, and TFQI relate to executive function. The strongest relationships were observed between thyroid hormones and executive function, with body composition showing limited moderating effects. In contrast, body composition—particularly weight and waist circumference—significantly moderates the relationship between thyroid hormones and short-term and delayed memory. These findings suggest that the association between thyroid hormones and cognitive function is influenced by body composition, highlighting the potential for weight management and abdominal fat reduction to improve cognitive outcomes. Overall, this study offers valuable insights into the mechanisms by which thyroid hormones affect brain function and provides guidance for clinical practice, particularly in optimizing cognitive health in relation to thyroid hormone function.

## Data Availability

The original contributions presented in the study are included in the article/**Supplementary Material**. Further inquiries can be directed to the corresponding author.
