# Supplementary material for: Moderating effects of body composition biomarkers on the relationship between thyroid hormones and cognitive performance in euthyroid older adults: insights from NHANES data
Source: Front Endocrinol (Lausanne). 2024 Nov 21;15:1487614. doi: 10.3389/fendo.2024.1487614 (PMC11617516; doi:10.3389/fendo.2024.1487614)
Supplement: Supplementary file 1 [file DataSheet1.pdf]

SUPPLEMENTARY

- Delimitation
- Regression Analyses of Thyroid Hormones Predicting Cognitive Function
- References

Delimitations

1. **FT3** is the unbound portion of triiodothyronine secreted by the thyroid gland, the most biologically active thyroid hormone responsible for regulating metabolism, cardiac function, and temperature balance (1). In this study, FT3 was measured in pg/mL.
2. **FT4** is the unbound portion of thyroxine secreted by the thyroid gland, primarily responsible for regulating metabolic activity by converting to triiodothyronine. It is an important indicator of hypothyroidism (1). FT4 was measured in ng/dL.
3. **TT3** includes both free and bound triiodothyronine in the blood and is age-related, making it the most sensitive indicator of hyperthyroidism (2). TT3 was measured in ng/dL.
4. **TT4** is the primary product secreted by the thyroid gland, encompassing both free and bound thyroxine in the blood (3). TT4 levels are used to assess hyperthyroidism, primary and secondary hypothyroidism (1). TT4 was measured in ug/dL.
5. **TG** is a protein produced by thyroid follicular cells, serving as a precursor to T3 and T4. It is one of the most commonly used indicators for monitoring the recurrence and metastasis of differentiated thyroid cancer (4). TG was measured in ng/mL.
6. **TGAb** is an autoantibody against thyroglobulin, present in certain autoimmune thyroid diseases (e.g., Hashimoto's thyroiditis and Graves' disease; 4). TGAb was measured in IU/mL.
7. **TSH**, secreted by the anterior pituitary gland, regulates thyroid function by stimulating the secretion of T3 and T4 (3). Elevated TSH levels indicate hypothyroidism, while decreased levels are common in hyperthyroidism. TSH is one of the most used indicators in thyroid function assessment. TSH was measured in uIU/mL.

8. **TPOAb** is an autoantibody against thyroid peroxidase, often elevated in thyroiditis caused by autoimmune diseases, especially Hashimoto's thyroiditis (5). TPOAb was measured in IU/mL.
9. The **FT3/FT4 ratio** reflects deiodinase activity and peripheral sensitivity to thyroid hormones, capable of predicting the risk of various chronic diseases (e.g., insulin resistance, metabolic syndrome, cardiometabolic dysfunction, and hyperthyroidism; 6). It is also a sensitive indicator for assessing metabolic health when thyroid function is otherwise normal (7). In this study, FT3/FT4 was calculated using pg/mL and ng/dL, with a higher FT3/FT4 ratio indicating higher peripheral thyroid hormone sensitivity. The formula is:

$$\text{FT3/FT4 ratio} = \text{FT3}\ (pg/mL)\ /\text{FT4 (ng/dL)}\ (8)$$

10. **TFQI** is a new indicator reflecting central sensitivity to thyroid hormones (2). The formula is:

$$TFQI = cdfFT4 - (1 - cdfTSH)\ (9)$$

11. **TSHI** is also an indicator of central sensitivity to thyroid hormones, with higher values indicating decreased central sensitivity to thyroid hormones (8). The formula is:

$$TSHI = Ln\, TSH\ (mIU/L) + 0.1345 \times FT4\ (pmol)\ (9)$$

12. **TT4RI** is a structured parameter used to calculate thyroid homeostasis, primarily for assessing indications of thyroid-stimulating hormone and non-thyroid diseases (8). The formula is:

$$TT4RI = FT4\ (pmol/L) \times TSH\ (mIU/L)\ (10)$$

Regression Analyses of Thyroid Hormones Predicting Cognitive Function

1. Short-term memory

Table S1.

Logistic Regression Analysis of TT3 Predicting Short-Term Memory

		Model 1	Model 2
Predictor	TT3	2.11 (0.035)	1.57 (0.116)
	Age		-3.80 (0.001)
	Sex		4.33 (0.001)
	Education		2.91 (0.004)
	Level 2		
	3		4.15 (0.001)
	4		4.70 (0.001)
	5		6.43 (0.001)
Pseudo R^2 (p)		0.003 (0.034)	0.047 (0.001)

Note. Model 1 includes only TT3 as the predictor, with short-term memory as the outcome variable; Model 2 adjusts for Age, Sex, and education level as confounding factors in the original model. Education level is coded with junior high school or below (1) as the reference category, ranging from low to high (2 to 5).

Table S2.

Logistic Regression Analysis of TSHI Predicting Short-Term Memory

		Model 1	Model 2
Predictor	TSHI	-2.38 (0.017)	-1.15 (0.251)
	Age		-3.92 (0.001)
	Sex		4.25 (0.001)
	Education		2.80 (0.005)
	Level 2		
	3		4.17 (0.001)
	4		4.64 (0.001)
	5		6.27 (0.001)
Pseudo R^2 (p)		0.003 (0.017)	0.047 (0.001)

Note. Model 1 includes only TSHI as the predictor, with short-term memory as the outcome variable; Model 2 adjusts for Age, Sex, and education level as confounding factors in the original model. Education level is coded with junior high school or below (1) as the reference category, ranging from low to high (2 to 5).

Table S3.

Logistic Regression Analysis of TFQI Predicting Short-Term Memory

		Model 1	Model 2
Predictor	TSHI	-1.91 (0.056)	-1.10 (0.269)
	Age		-4.01 (0.001)
	Sex		4.30 (0.001)
	Education		2.82 (0.005)
	Level 2		
	3		4.15 (0.001)
	4		4.66 (0.001)
	5		6.30 (0.001)
Pseudo R^2 (p)		0.002 (0.055)	0.046 (0.001)

Note. Model 1 includes only TFQI as the predictor, with short-term memory as the outcome variable; Model 2 adjusts for Age, Sex, and education level as confounding factors in the original model. Education level is coded with junior high school or below (1) as the reference category, ranging from low to high (2 to 5).

2. Delayed memory

Table S4.

Logistic Regression Analysis of FT3 Predicting Delayed Memory

		Model 1	Model 2
Predictor	FT3	2.97 (0.003)	2.40 (0.017)
	Age		-5.31 (0.001)
	Sex		4.84 (0.001)
	Education		2.40 (0.016)
	Level 2		
	3		4.33 (0.001)
	4		5.22 (0.001)
	5		6.46 (0.001)
Pseudo R^2 (p)		0.004 (0.003)	0.059 (0.001)

Note. Model 1 includes only FT3 as the predictor, with delayed memory as the outcome variable; Model 2 adjusts for Age, Sex, and education level as confounding factors in the original model. Education level is coded with junior high school or below (1) as the reference category, ranging from low to high (2 to 5).

Table S5.

Logistic Regression Analysis of TT3 Predicting Delayed Memory

		Model 1	Model 2
Predictor	TT3	3.36 (0.001)	2.44 (0.015)
	Age		-5.20 (0.001)
	Sex		4.63 (0.001)
	Education		2.28 (0.017)
	Level 2		
	3		4.29 (0.001)
	4		5.25 (0.001)
	5		6.52 (0.001)
Pseudo R^2 (p)		0.006 (0.001)	0.059 (0.001)

Note. Model 1 includes only TT3 as the predictor, with delayed memory as the outcome variable; Model 2 adjusts for Age, Sex, and education level as confounding factors in the original model. Education level is coded with junior high school or below (1) as the reference category, ranging from low to high (2 to 5).

Table S6.

Logistic Regression Analysis of TSHI Predicting Delayed Memory

			Model 1	Model 2
Predictor	TSHI		-2.59 (0.010)	-1.16 (0.248)
	Age			-5.72 (0.001)
	Sex			4.54 (0.001)
	Education			2.36 (0.018)
	Level 2			
		3		4.20 (0.001)
		4		5.11 (0.001)
		5		6.31 (0.001)
Pseudo R^2 (p)			0.003 (0.010)	0.057 (0.001)

Note. Model 1 includes only TSHI as the predictor, with delayed memory as the outcome variable; Model 2 adjusts for Age, Sex, and education level as confounding factors in the original model. Education level is coded with junior high school or below (1) as the reference category, ranging from low to high (2 to 5).

3. Executive function

Table S7.

Linear Regression Analysis of FT4 Predicting Executive Function

		Model 1	Model 2
Predictor	FT4	-2.99 (0.003)	-2.70 (0.007)
	Age		-4.67 (0.001)
	Sex		-1.19 (0.233)
	Education		0.86 (0.390)
	Level 2		
	3		2.85 (0.005)
	4		4.45 (0.001)
	5		7.40 (0.001)
Pseudo R^2 (p)		0.018 (0.003)	0.194 (0.001)

Note. Model 1 includes only FT4 as the predictor, with executive function as the outcome variable; Model 2 adjusts for Age, Sex, and education level as confounding factors in the original model. Education level is coded with junior high school or below (1) as the reference category, ranging from low to high (2 to 5).

Table S8.

Linear Regression Analysis of FT3/FT4 Ratio Predicting Executive Function

		Model 1	Model 2
Predictor	FT3/FT4 ratio	2.91 (0.004)	2.24 (0.025)
	Age		-4.40 (0.001)
	Sex		-1.06 (0.289)
	Education		0.83 (0.406)
	Level 2		
	3		2.93 (0.004)
	4		4.48 (0.001)
	5		7.43 (0.001)
Pseudo R^2 (p)		0.017 (0.004)	0.190 (0.001)

Note. Model 1 includes only FT3/FT4 ratio as the predictor, with executive function as the outcome variable; Model 2 adjusts for Age, Sex, and education level as confounding factors in the original model. Education level is coded with junior high school or below (1) as the reference category, ranging from low to high (2 to 5).

Table S9.

Linear Regression Analysis of TSHI Predicting Executive Function

		Model 1	Model 2
Predictor	TSHI	-2.29 (0.023)	-1.49 (0.137)
	Age		-4.46 (0.001)
	Sex		1.32 (0.188)
	3		2.91 (0.004)
	4		4.41 (0.001)
	5		7.29 (0.001)
Pseudo R^2 (p)		0.010 (0.023)	0.185 (0.001)

Note. Model 1 includes only TSHI as the predictor, with executive function as the outcome variable; Model 2 adjusts for Age, Sex, and education level as confounding factors in the original model. Education level is coded with junior high school or below (1) as the reference category, ranging from low to high (2 to 5).

Table S10.

Linear Regression Analysis of TFQI Predicting Executive Function

		Model 1	Model 2
Predictor	TFQI	-2.52 (0.012)	-1.99 (0.047)
	Age		-4.47 (0.001)
	Sex		-1.30 (0.193)
	Education		0.69 (0.489)
	Level 2		
	3		2.90 (0.004)
	4		4.45 (0.001)
	5		7.32 (0.001)
Pseudo R^2 (p)		0.012 (0.012)	0.188 (0.001)

Note. Model 1 includes only TFQI as the predictor, with executive function as the outcome variable; Model 2 adjusts for Age, Sex, and education level as confounding factors in the original model. Education level is coded with junior high school or below (1) as the reference category, ranging from low to high (2 to 5).

References

1. Nauman JA, Nauman A, Werner SC, others. Total and free triiodothyronine in human serum. *The Journal of clinical investigation* (1967) 46:1346–1355.
2. Ding X, Wang Y, Liu J, Wang G. Impaired Sensitivity to Thyroid Hormones Is Associated With Elevated Homocysteine Levels in the Euthyroid Population. *The Journal of Clinical Endocrinology* (2022)
3. Van Uytfanghe K, Ehrenkranz J, Halsall D, Hoff K, Loh TP, Spencer CA, Köhrle J, Group ATFTW. Thyroid stimulating hormone and thyroid hormones (triiodothyronine and thyroxine): an American Thyroid Association-commissioned review of current clinical and laboratory status. *Thyroid* (2023) 33:1013–1028.
4. Spencer C, Fatemi S. Thyroglobulin antibody (TgAb) methods–strengths, pitfalls and clinical utility for monitoring TgAb-positive patients with differentiated thyroid cancer. *Best practice & research Clinical endocrinology & metabolism* (2013) 27:701–712.
5. Takasu N, Yoshimura Noh J. Hashimoto's thyroiditis: TGAb, TPOAb, TRAb and recovery from hypothyroidism. *Expert Review of Clinical Immunology* (2008) 4:221–237.
6. Štěpánek L, Horáková D, Štěpánek L, Janout V, Janoutová J, Bouchalová K, Martiník K. Free triiodothyronine/free thyroxine (FT3/FT4) ratio is strongly associated with insulin resistance in euthyroid and hypothyroid adults: a cross-

sectional study. *Endokrynologia Polska* (2021) 72:8–13.

7. Lang X, Li Y, Zhang D, Zhang Y, Wu N, Zhang Y. FT3/FT4 ratio is correlated with all-cause mortality, cardiovascular mortality, and cardiovascular disease risk: NHANES 2007-2012. *Frontiers in Endocrinology* (2022) 13:964822.
8. Yu Z-W, Pu S-D, Sun X-T, Wang X-C, Gao X-Y, Shan Z-Y. Impaired sensitivity to thyroid hormones is associated with mild cognitive impairment in euthyroid patients with type 2 diabetes. *Clinical interventions in aging* (2023)1263–1274.
9. Jostel A, Ryder WDJ, Shalet SM. The use of thyroid function tests in the diagnosis of hypopituitarism: definition and evaluation of the TSH Index. *Clinical endocrinology* (2009) 71:529–534.
10. Yagi H, Pohlenz J, Hayashi Y, Sakurai A, Refetoff S. Resistance to thyroid hormone caused by two mutant thyroid hormone receptors β, R243Q and R243W, with marked impairment of function that cannot be explained by altered in vitro 3, 5, 3′-triiodothyroinine binding affinity. *The Journal of Clinical Endocrinology & Metabolism* (1997) 82:1608–1614.
